# Longitudinal alterations in morphological brain networks and cognitive function in common-type COVID-19: a 3-month follow-up study

**DOI:** 10.3389/fneur.2025.1549195

**Published:** 2025-04-15

**Authors:** Ying Liu, Bei Peng, Haixia Qin, Kaixuan Zhou, Shihuan Lin, Yinqi Lai, Lingyan Liang, Gaoxiong Duan, Xiaocheng Li, Xiaoyan Zhou, Yichen Wei, Qingping Zhang, Jinli Huang, Yan Zhang, Jiazhu Huang, Ruijing Sun, Sijing Tuo, Yuxin Chen, Demao Deng

**Affiliations:** ^1^Department of Radiology, The First Affiliated Hospital of Jinan University, Guangzhou, China; ^2^Department of Radiology, The People’s Hospital of Guangxi Zhuang Autonomous Region, Guangxi Academy of Medical Sciences, Nanning, China

**Keywords:** graph theory, morphological brain network, COVID-19, cognitive function, magnetic resonance imaging

## Abstract

**Purpose:**

To investigate the morphological network and cognitive function of patients with common-type coronavirus disease 2019 (COVID-19) during the acute phase, and examine dynamic changes at 3-month follow-up.

**Methods:**

At baseline, high-resolution T1-weighted imaging was conducted in 35 patients with COVID-19 and 40 healthy controls; 22 patients were reassessed at 3 months. All patients underwent cognitive assessments. Individual morphological brain networks were constructed using grey matter volume similarity, and topological properties were analyzed using graph theory. We used an independent sample *t*-test at baseline and a paired sample *t*-test to compare the 3-month follow-up with the acute phase, with false discovery rate corrections (*p* < 0.05).

**Results:**

In the acute phase, patients exhibited increased subcortical network (SCN) connectivity, and reduced connectivity between the frontoparietal network (FPN) and limbic network (LN), the SCN and dorsal/ventral attention network (DAN/VAN), and the LN and DAN. At follow-up, SCN connectivity remained elevated, with partial recovery in SCN-DAN/VAN and LN-DAN connectivity, and significant FPN-LN improvements. Enhanced global efficiency and reduced path length indicated improved network integration. Additionally, digit symbol substitution test and verbal fluency test scores improved over time.

**Conclusion:**

COVID-19 induces short-term disruptions in cognition-related morphological subnetworks, with subcortical networks compensating for these changes. Significant recovery in FPN-LN connectivity and partial restoration of other networks highlight the plasticity of the brain and suggest that FPN-LN connectivity is a potential neuroimaging marker for cognitive recovery.

## 1 Introduction

The 2019 coronavirus outbreak, which caused severe acute respiratory syndrome (SARS-CoV-2), had a profound global impact owing to its high contagiousness and rapid spread worldwide. Beyond respiratory symptoms, increasing evidence indicates that the virus can induce a range of neurological symptoms in patients with coronavirus disease 2019 (COVID-19), including cognitive impairments, such as inattention, memory loss, and slowed processing speed (1–6). These symptoms can persist long after the acute phase, significantly affecting recovery. A deeper understanding of these neurological manifestations and their underlying etiologies is crucial for effective intervention.

Magnetic resonance imaging (MRI) studies have provided critical insights into COVID-19’s impact on brain structure. Alterations in white matter volume, signal abnormalities, and grey matter (GM) volume have been documented in patients with COVID-19 (7–10). Furthermore, longitudinal studies have revealed persistent white matter damage and structural abnormalities in the supraoccipital gyrus at 3-month follow-up (11). Abnormal GM network patterns observed during this period also suggest structural covariance disruptions, potentially linked to neurological symptoms, such as headaches (12).

Morphological brain networks (MBNs) offer a powerful framework for analyzing spatial similarities in GM across brain regions. By leveraging techniques such as information theory, MBNs enable cost-effective, large-scale studies with high-resolution insights (13, 14). Despite their widespread application in the study of neurological disorders, such as Parkinson’s and Alzheimer’s disease (15–17), MBNs have seen limited application in COVID-19 research. Graph theory further enhances brain network analysis by quantifying structural and functional connectivity, capturing global configurations, neural hubs, and modular structures (18, 19). Notably, studies have identified reduced global and local network efficiency in patients with COVID-19, underscoring the need for further research into the virus’s neurological impact (20).

To this end, this study focused on constructing individual-level MBNs and performing cognitive assessments in patients with common-type COVID-19 during the acute phase and at 3-month follow-up. This study aimed to examine the abnormalities in morphological network connectivity and properties observed in COVID-19 during the acute phase, their dynamic changes over the follow-up period, and the extent of recovery. Additionally, we focus on exploring the potential mechanisms of network changes and evaluated their associations with cognitive function. The outcomes of this study may provide a foundation for investigating the long-term effects of COVID-19.

## 2 Materials and methods

### 2.1 Ethics statement

The study adhered to the Declaration of Helsinki and received approval from the Medicine Ethics Committee of the People’s Hospital of the Guangxi Zhuang Autonomous Region (Approval No. KY-IIT-2022-05). All participants provided informed consent, and the study was registered with the Chinese Clinical Trial Registry (ChiCTR2300071002).

### 2.2 Participants

This study used a longitudinal design. Patients enrolled in the acute phase between December 2022 and January 2023, and returning patients who recovered 3 months later were included in this trial. According to the guidelines of the National Health Commission (8th edition, in Chinese), we selected patients with common type COVID-19 characterized by pneumonia on computed tomography, with attendant fever and respiratory symptoms. In the acute phase, which occurs within the first 1–2 weeks of COVID-19 infection, 35 patients with common-type COVID-19 were enrolled, 22 of whom agreed to undergo a 3-month follow-up survey. All patients underwent face-to-face psychiatric assessments and MRI was conducted by trained medical personnel. A control group of healthy participants, which were obtained from previous research (Supplementary material S1), was matched to the patient group by age and sex. An identical MRI methodology was used for healthy controls, during which no one reported any history of COVID-19, neurological, or cognitive disorder.

### 2.3 MRI acquisition

Each participant underwent high-resolution 3D T1-weighted imaging (3D T1WI) using a Siemens Magnetom Vida 3.0 T MRI scanner. The imaging parameters were as follows: repetition time (TR) of 2,000 ms, echo time (TE) of 2.02 ms, matrix size of 256 × 256, flip angle of 7°, and slice thickness of 1 mm.

### 2.4 Cognitive assessment acquisition

All patients with common COVID-19 completed the following five cognitive assessments: (1) the logical memory (LM) test, which evaluates verbal episodic memory. Participants were required to repeat a sentence immediately (LM-A) after hearing it and after a 30-min delay (LM-B). (2) The digit span task, which assesses verbal attention and working memory through forward digit span and backward digit span tasks that evaluate visual and visuospatial sequence representations, respectively. Digits with lengths from 2 to 9 were presented in increasing order. (3) The digit symbol substitution test (DSST) is widely utilized to assess processing speed, sustained attention, and working memory. Participants had 2 min to match nine numbers with their corresponding symbols. Higher scores indicated superior performance. (4) The Knowledge subscale of the Wechsler Intelligence Scale assesses participants’ breadth of knowledge, learning capacity, and comprehension of everyday phenomena. Common-sense questions were asked, such as identifying the season with the longest days or the time of day with the shortest shadows. (5) Participants in the verbal fluency test (VFT) were tasked with listing as many animals as they could in 1 min. Neuropsychological tests and MRI scans were conducted on the same day.

### 2.5 Data analysis

#### 2.5.1 MRI preprocessing

T1-weighted images underwent voxel-based morphometry analysis using the Computational Anatomy Toolbox (CAT12, http://www.neuro.uni-jena.de/cat/) integrated with Statistical Parametric Map 12 (SPM12, http://www.fil.ion.ucl.ac.uk/spm/software/spm12/) for voxel-based morphometry analysis. This process aims to derive voxel-wise GM volumes for all participants. Initially, T1-weighted images from all participants underwent bias-field inhomogeneity correction, followed by segmentation into GM, white matter, and cerebrospinal fluid. The resulting GM images were spatially normalized using the DARTEL algorithm aligned to the Montreal Neurological Institute space. Nonlinear modulation alleviated the impact of spatial normalization, while repeated alignment created a GM template for the entire cohort. Subsequently, a 6-mm full width at half maximum was used to smooth the scans. Each participant’s GM volume map was generated post-preprocessing with a voxel size of 1.5 × 1.5 × 1.5 mm^3^.

#### 2.5.2 Brain parcellation

The Brainnetome Atlas segmented the brain into 246 regions of interest (ROIs) to establish network nodes (21). The constructed networks were classified into eight regions: the dorsal attention network (DAN), default mode network (DMN), frontoparietal network (FPN), limbic network (LN), subcortical network (SCN), somatomotor network (SMN), ventral attention network (VAN), and visual network (VN). In this study, we focused exclusively on six cognition-related networks: DAN, DMN, FPN, LN, SCN, and VAN.

#### 2.5.3 Construction of individual MBN

**Figure 1:**
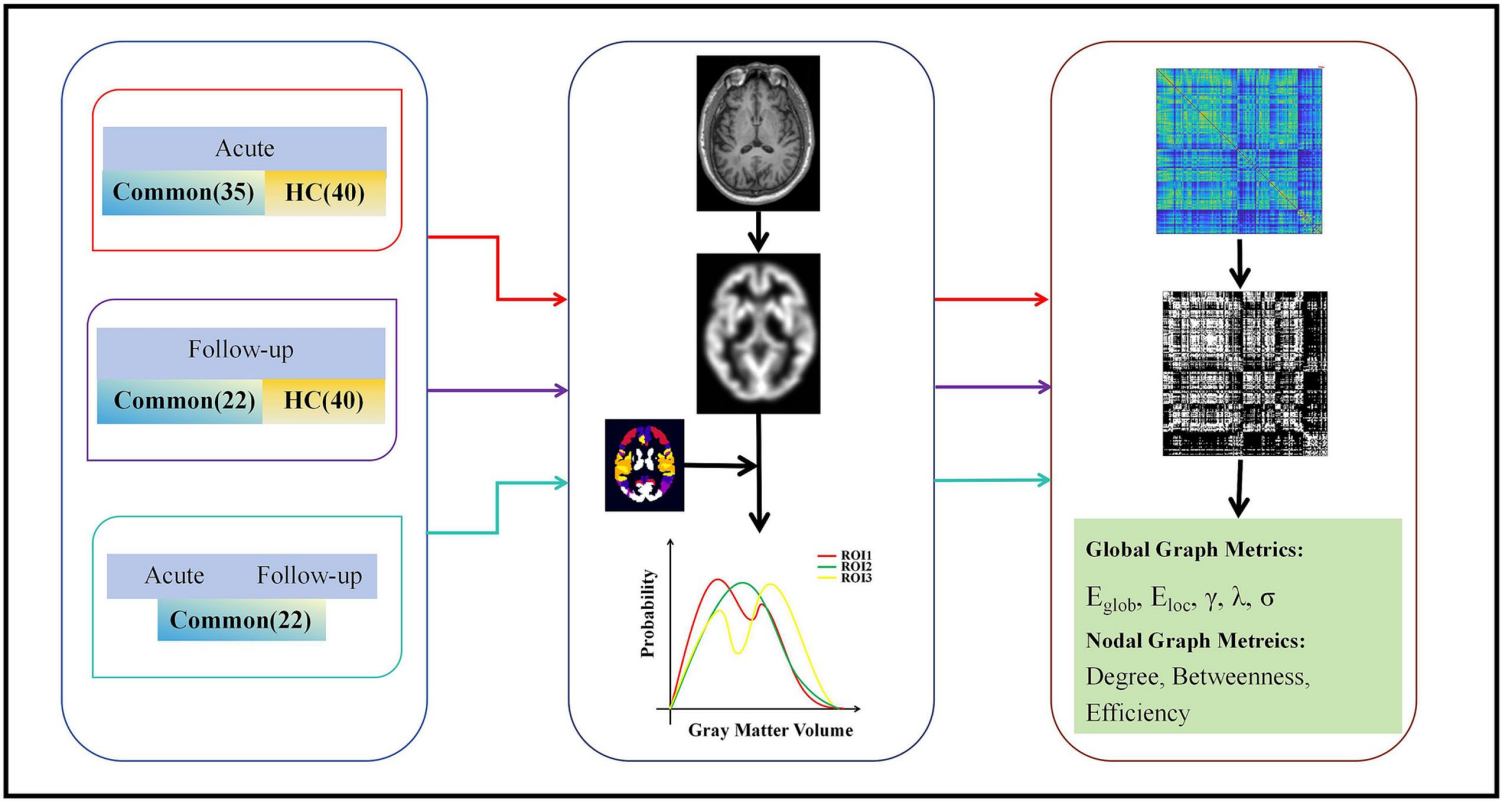
Flow chart for the construction of morphological networks and graph theory analysis. Individual T1 images were segmented to reconstruct the center surface and obtain morphometric maps, and then each morphometric map was divided into 246 cortical blocks based on the Human Brain atlas. Morphological indices were extracted separately for each region to estimate the probability density function. The KLD between the probability density functions of each pair of regions was calculated to form a morphological similarity network matrix. This matrix was then analyzed using the graph theory. *E*_g_, global efficiency; *E*_loc_, local efficiency; *λ*, normalized feature path length; *γ*, normalized clustering coefficient; *σ*, small-worldness; KLD, Kullback–Leibler divergence.

Large-scale MBNs for each participant were constructed based on the GM volumes, as depicted in Figure 1. In this context, a brain network comprises nodes and edges, where nodes correspond to specific brain regions, and edges signify morphological distribution similarity. Individual morphological similarity networks were created using similarity measures, and inter-regional similarity was evaluated through Kullback–Leibler divergence-based similarity (KLDs) (13, 14). Probability density estimates were subsequently computed for each ROI and morphological index were calculated using a normal kernel function. Subsequently, the resulting probability density function was altered into a probability distribution function (PDF). The KLD between PDFs *P* and *Q* was computed using the following method:


(1) $$\mathrm{KLDs}\;\left(P,Q\right)=e^{-D_{KL}\left(P,Q\right)}\text{,}$$

A metric ranging from 0 to 1 was derived, where 0 signifies maximum separability between the GM *P* and *Q* density distributions, and 1 denotes two identical distributions. Equation 1 is based on the Kullback–Leibler divergence (*D*_KL_), which is calculated as shown in Equation 2:


(2) $$D_{KL}\;\left(P,Q\right)=\overset{n}{\underset{i=1}{\sum }}\left(P\left(i\right)\log\;\frac{P\left(i\right)}{Q\left(i\right)}+Q\left(i\right)\log\;\frac{Q\left(i\right)}{P\left(i\right)}\right)\text{,}$$

where *n* represents the total count of sampling points. The KLD values were computed for all pairs of brain regions to create a 246 × 246 whole-brain MBN (KLDS-based morphological connection matrix) for each participant.

#### 2.5.4 Graph-theory analysis

All morphological network analyses were performed using Graph Theoretical Network Analysis Toolbox (GRETNA, https://www.nitrc.org/projects/gretna/), a MATLAB-based software for brain connectome analysis (22). Sparsity thresholds were selected using the following criteria: minimal sparsity was chosen to ensure that the average degree over all nodes of each network was more extensive than log (*N*).The maximal sparsity was selected to ensure that each threshold network’s small-worldness scalar (sigma) was more extensive than 1.1. Global network metrics included network integration and segregation measures. For network integration, we focused on the global efficiency (*E*_glob_) and normalized characteristic path length (*λ*). Regarding network segregation, we examined the local efficiency (*E*_loc_) and normalized clustering coefficient (*γ*). Additionally, we assessed the small-worldness measure (*σ*) to completely evaluate the characteristics of the network (23, 24). We evaluated each network metric by calculating the area under the curve across various S thresholds, providing a comprehensive summary of GM morphological network properties (25).

We characterized node characteristics in the morphological network using three key parameters: nodal betweenness centrality, nodal degree centrality, and nodal efficiency. Nodal betweenness quantifies a node’s role as an intermediary on the shortest paths between other nodes, indicating its capacity to regulate information flow within the network (26). Nodal degree quantifies a node’s direct connections, indicating its extensive network connectivity (27). Nodal efficiency quantifies the effectiveness of information transmission a network. It is numerically defined as the inverse of the average shortest path length from one node to all other nodes (28).

### 2.6 Statistical analysis

Statistical analyses were conducted using MATLAB 2016 (MathWorks Inc., MA) or SPSS version 21.0 software (IBM Corp, Armonk, New York). The *t*-test was applied to quantitative variables, while the chi-square test was used for qualitative variables. A two-sample *t*-test was used to compare the demographic and clinical data of patients with common-type COVID-19 and healthy controls. For comparison between the acute phase and follow-up trials, paired *t*-tests were used. A *p*-value threshold of 0.05 was used for statistical significance, with FDR correction.

## 3 Results

### 3.1 Demographic and clinical characteristics

This study included 35 patients in the common-type group during the acute phase, 22 of whom were followed up for 3 months. Additionally, 40 HCs were enrolled. Table 1 presents the clinical and demographic characteristics of the participants. No statistically significant differences in age or sex were observed between the common-type group and the HC group, either during the acute phase or at the 3-month follow-up.

**Table 1:** Demographic and clinical characteristics.

Characteristic	Acute			Follow-up		
	Common (*n* = 35)	HC (*n* = 40)	*p*-value	Common (*n* = 22)	HC (*n* = 40)	*p*-value
Age (years)	54.3 ± 13.8	47.6 ± 17.5	0.072^a^	52.4 ± 14.5	47.6 ± 17.5	0.273^a^
Sex (F/M)	23/12	18/22	0.07^b^	14/8	18/22	0.16^b^

Data are expressed as mean ± standard deviation unless otherwise stated. *p*^a^ denotes the *p*-value from the two-sample *t*-test, and *p*^b^ denotes the *p*-value from the chi-square test. F, female; M, male.

Notably, after 3 months, the DSST and VFT scores of the common group significantly increased compared to those in the acute phase (*p* < 0.05), as shown in Table 2.

**Table 2:** Changes in neuropsychological test scores at baseline and follow-up.

Characteristic	Acute common (*n* = 22)	Follow-up common (*n* = 22)	*p*-value
LM	13.4 ± 9.4	13.2 ± 8.9	0.945^c^
DS	19.9 ± 5.3	20.7 ± 4.6	0.242^c^
DSST	47.2 ± 20.5	55.5 ± 21.1	0.005^c^
The Knowledge subscale of the Wechsler Intelligence Scale	17.8 ± 5.3	19.2 ± 5.8	0.093^c^
VFT	14.7 ± 5.9	17.1 ± 5.8	0.002^c^

*pc denotes the p-value of the paired samples t-test. DS, digit span; DSST, digital symbol substitution test; LM, logical memory; VFT, verbal fluency test.*

### 3.2 Alterations of morphological connection strength

To better account for the variations in subnetwork-level morphological connectivity between the common-type and HC groups, we examined the intensity of morphological connectivity within and between networks.

**Figure 2:**
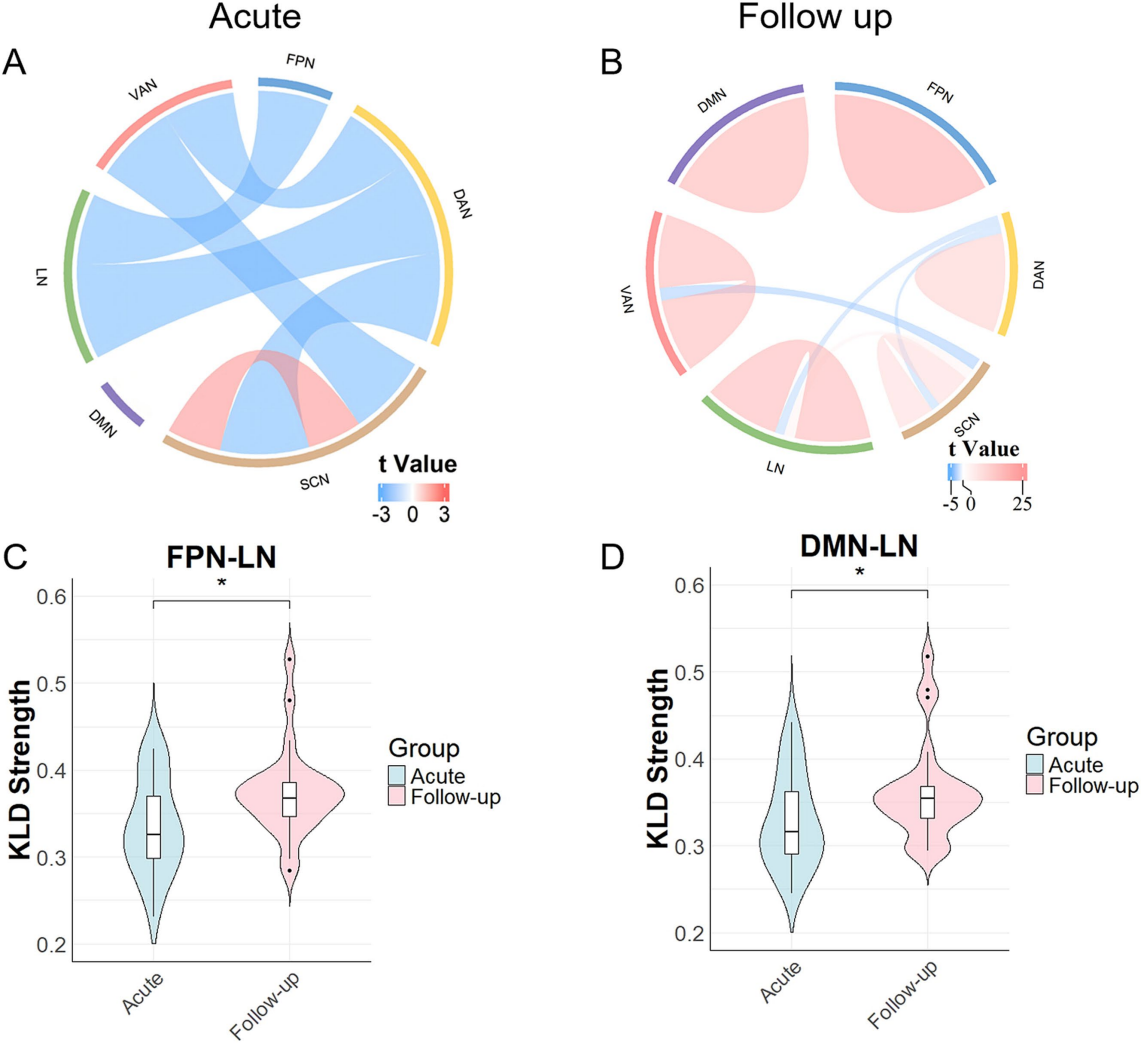
Results of the comparison of morphological network strengths. **(A)** Morphological network changes during the acute phase in the common group compared with those in the healthy control (HC) group. Network connectivity within the SCN increased, whereas network connectivity between the SCN-DAN, SCN-VAN, LN-DAN, FPN-LN, and DAN-VAN decreased. **(B)** Changes at the 3-month follow-up, with increased network connectivity within the SCN, DMN, DAN, VAN, LN, and FPN, as well as between the SCN-LN, and decreased network connectivity between the SCN-DAN, SCN-VAN, and LN-DAN. **(C)** Network connectivity between FPN-LNs in the common-type group significantly increased after 3 months compared to that in the acute phase. **(D)** Network connectivity between DMN-LNs in the common-type group significantly increased after 3 months compared to that in the acute phase. *p < 0.05. DAN, dorsal attention network; DMN, default mode network; FPN, frontoparietal network; LN, limbic network; SCN, subcortical network; VAN, ventral attention network.

In the acute phase, compared with the HC group, the common-type group exhibited significantly higher morphological connectivity within the SCN, whereas connectivity between SCN-DAN, SCN-VAN, LN-DAN, and DAN-VAN was reduced. Additionally, the connectivity between FPN-LN decreased, with all differences being statistically significant, as illustrated in Figure 2A and Supplementary material S2.

After 3 months, the common-type group continued to show increased connectivity within the SCN, and decreased connectivity between the SCN-DAN, SCN-VAN, and LN-DAN groups compared to those in the HC group. Connectivity within the FPN, LN, DMN, DAN, and VAN also increased with SCN-LN connectivity, all of which were statistically significant (Figure 2B and Supplementary Table S1). Notably, as shown in Figure 2C, FPN-LN connectivity in the common-type group was significantly increased compared to that in the acute phase (*p* < 0.05), whereas it was not significantly different compared with the HC group. Additionally, as shown in Figure 2D, DMN-LN connectivity was also significantly increased compared to that in the acute phase (*p* < 0.05). No significant statistical differences were observed between the DMN and other networks, regardless of whether it was during the acute phase or at the 3-month follow-up.

### 3.3 Alterations of global brain network metrics

**Figure 3:**
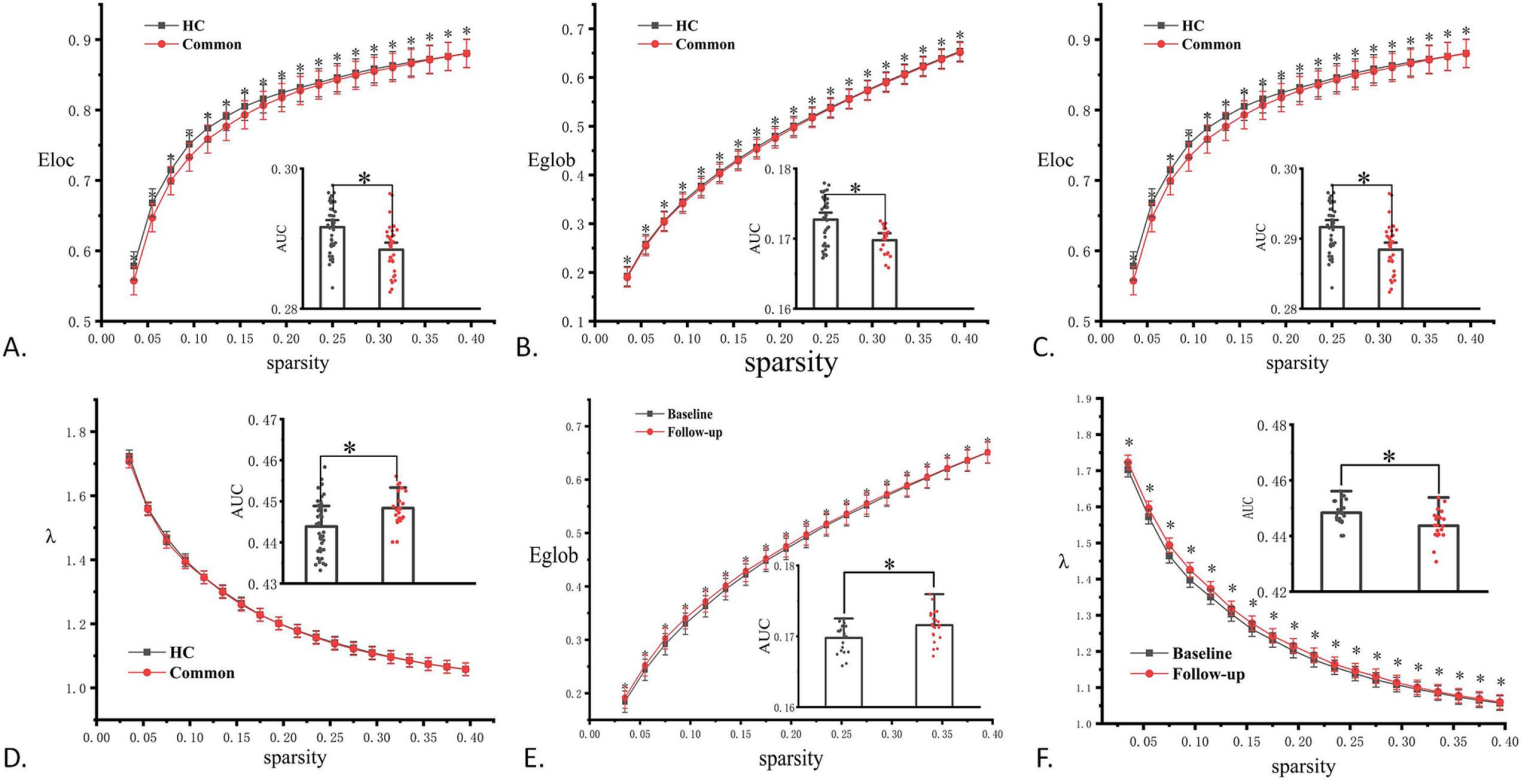
Comparison results for topological properties. **(A)** Significant differences in local efficiency (*E*_loc_) in the common group during the acute phase compared to that in the HC group. **(B–D)** Significant differences in global efficiency (*E*_g_), clustering coefficient (*λ*), and local efficiency (*E*_loc_) in the common group at the 3-month follow-up compared to that in the HC group. **(E, F)** Significant differences in *E*_g_ and *λ* in the regular group during follow-up compared to that in the acute phase. *p < 0.05. AUC, area under the curve; HC, healthy control.

During the acute phase, as shown in Figure 3A, the common-type group showed a significant difference in *E*_loc_ compared to that in the HC group, with a decreasing trend. After 3 months, compared to the HC group, the common-type group showed significant differences in *E*_g_, *λ*, and *E*_loc_, with *E*_g_ and *E*_loc_ showing a decreasing trend and *λ* showing an increasing trend, as shown in Figures 3B–D. The common-type group showed a significant increase in *E*_g_ and a significant decrease in *λ* at follow-up compared to the acute phase, as illustrated in Figures 3E,F.

### 3.4 Alterations in node properties

**Figure 4:**
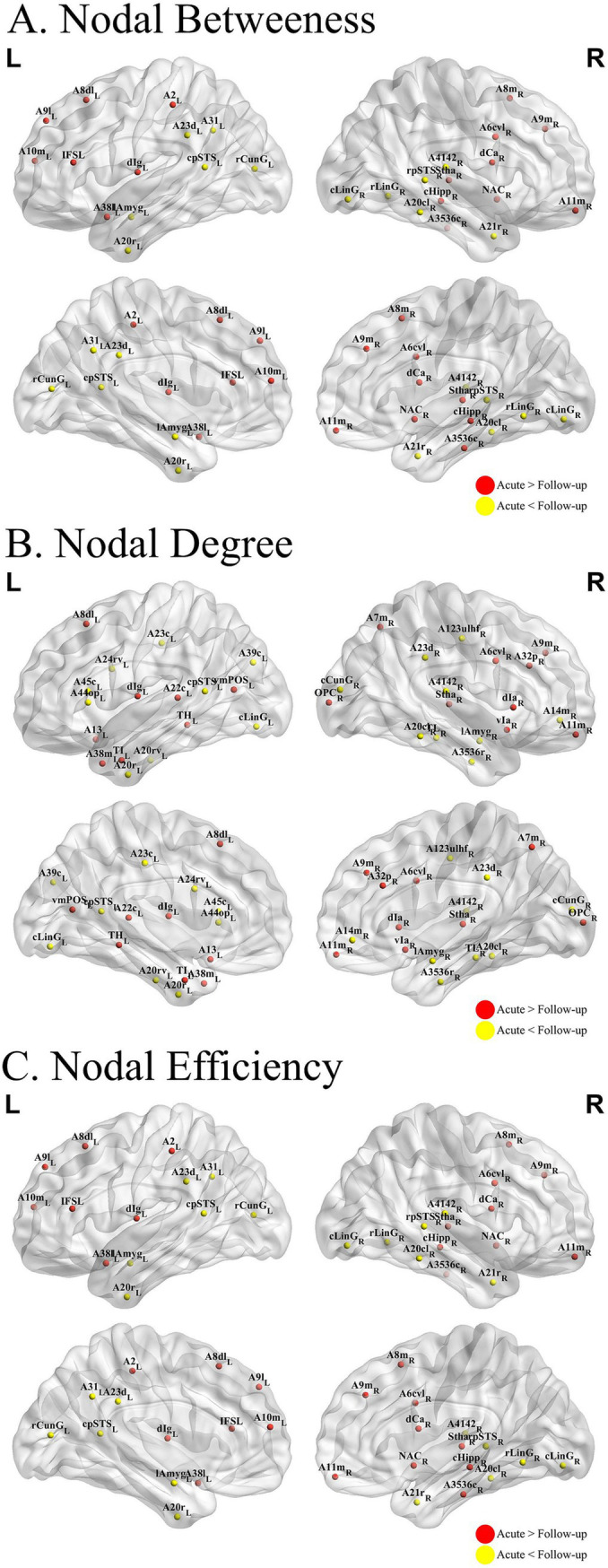
Nodal analysis results. ALTERATIONS in structural network topologies in patients with common-type COVID-19 **(A–C)** Group differences in betweenness **(A)**, degree **(B)**, and efficiency **(C)** at nodal level. The KLDs-based network showed that compared to the acute phase, patients in the follow-up phase had altered node profiles in some specific regions, mainly located in the frontal lobe, temporal lobe, parietal lobe, and insula. COVID-19, coronavirus disease 2019.

Furthermore, we compared the node characteristics of the three individual MBNs in the common-type group during the acute and follow-up periods. The KLS-based network (Figure 4) showed that, compared with the acute phase, patients in the follow-up phase had altered node profiles in specific regions, mainly located in the frontal lobe, temporal lobe, parietal lobe, and insula, involving the FPN, VAN, and SCN.

## 4 Discussion

In this study, we used a morphological brain network to examine internetwork modifications and changes in network features in patients with common-type COVID-19 during the acute phase and at 3-month follow-up. The results revealed substantial alterations in morphological connectivity and network characteristics during both the acute phase and 3-month follow-up, compared to those in HCs. Significant differences in the FPN and LN were observed in patients at the follow-up relative to in the acute phase. However, network properties, including *E*_g_ and characteristic path length *λ*, exhibited opposing trends, with *E*_g_ increasing and *λ* decreasing. Neuropsychological testing revealed significant improvements in DSST and VFT scores between the pre- and post-follow-up comparisons, with higher scores at follow-up.

FPN plays a key role in higher cognitive functions, such as working memory, attention regulation, and executive functioning (29). Research suggests that anomalies in the FPN can result in deficits in executive function and cognitive control. These findings align with reports from patients regarding “brain fog,” memory loss, and difficulties in concentration (30). Moreover, studies have indicated that COVID-19 may lead to cognitive impairments in individuals during the acute phase, including attention difficulties, slow processing speed, and memory deficits (2). Additionally, compared to HCs, an increase in FPN subnetwork strength was observed in patients with COVID-19 during the follow-up period, which may indicate a gradual recovery of cognitive function over time. This is consistent with previous studies showing cognitive improvement in some patients with COVID-19 after rehabilitation, although long-term effects may persist (31). The LN is strongly associated with emotional regulation, memory, and autonomic functioning (32). Rogers et al. (33) have reported that patients with COVID-19 may experience emotional problems, such as anxiety, depression, and post-traumatic stress disorder, during the acute phase. Long-term follow-up studies have demonstrated a strong correlation between post-COVID syndrome-related depression and anxiety, and changes in the LN. This association may be explained by the structural changes caused by neuroinflammation in the hippocampus and amygdala (30). In addition, structural alterations in the LN may exacerbate difficulties in emotion regulation, making it more difficult for patients to recover from acute-phase trauma (34). Our findings revealed an increase in the LN subnetwork strength during the follow-up period, suggesting that, while the novel coronavirus may affect mood and emotional functioning, these emotional effects tend to gradually improve over time.

In this study, we primarily selected patients with common-type COVID-19, characterized by pulmonary inflammation, and conditions of inflammation and hypoxia. Physiologically and pathologically, the novel coronavirus may damage the central nervous system by directly infecting neurons or inducing a systemic inflammatory response (35). Inflammatory factors can disrupt the blood-brain barrier and compromise the integrity of brain networks, particularly the FPN and LN. Additionally, neuroimaging studies have demonstrated that GM thickness in patients with COVID-19 is altered after infection, particularly in regions related to cognition and emotion (30), supporting our findings that the FPN and LN are affected. During the acute phase of infection, a reduction in morphological connectivity between the FPN and LN was observed, which significantly increased after 3 months. This finding may reflect a reduction in the inflammatory response and the effects of neuroplasticity. However, we found no statistically significant difference in the FPN-LN connectivity of patients with COVID-19 and HCs after 3 months, suggesting that the morphological connectivity between the FPN and LN had largely returned to normal.

The DMN is critically involved in memory and language, with its effective suppression being essential for optimal cognitive performance (Zhou et al., 2016). Chang et al. (1) reported that patients with post-COVID-19 conditions (PCC) exhibited increased activation in the right superior frontal gyrus and attenuated deactivation in DMN regions during working memory tasks. This observation reflects neural compensation characterized by the reorganization of neural networks, and is associated with diminished cognitive function (1). In the present study, patients with mild COVID-19 demonstrated enhanced morphological connectivity within the DMN 3 months post-infection, suggesting incomplete cognitive recovery and ongoing inter-network remodeling. Li et al. (2023) identified a close relationship between cognitive impairment following COVID-19, alterations in DMN topology, and higher-order cognitive functions. Furthermore, Chen et al. (2023) revealed that morphological changes in the DMN-LN mediated the relationship between white matter hyperintensities and cognitive performance, particularly language function. In the current study, morphological connectivity between the DMN and LN was significantly increased 3 months post-COVID-19 compared to that during the acute phase. This enhancement aligns with observed improvements in cognitive and language functions, as evidenced by the significant increase in VFT scores.

Our study results showed that SCN morphological connectivity in patients with COVID-19 increased significantly during the acute phase compared with that in HCs and further intensified at the 3-month follow-up, indicating a compensatory role. The dynamic network model proposes that higher cognitive functions rely on a dynamic network that integrates the cortical and subcortical regions. Subcortical structures may compensate for cortical dysfunction, particularly in species with limited cortical tissue (36). Therefore, cortical dysfunction caused by COVID-19 may enhance the compensatory role of the subcortical network. Furthermore, we propose that the increased connectivity of the subcortical network may result from the promotion of neural activity in subcortical regions by inflammatory factors. COVID-19 infection may influence subcortical regions, such as the basal ganglia or thalamus, through the release of pro-inflammatory cytokines (e.g., interleukin-6, tumor necrosis factor-α). These regions, which are closely associated with emotion regulation and cognitive function, may exhibit “hyperactivity” or enhanced connectivity due to inflammation. Further, Mazza et al. (37) reported a relationship between neuroinflammation during the acute phase of COVID-19 and increased activity in subcortical network regions.

Our results indicate that the morphological connectivity between the SCN-VAN and SCN-DAN was significantly reduced during the acute phase. This aligns with a previous study, which showed that SARS-CoV-2 infection impairs attention and leads to cognitive decline by disrupting functional connectivity between subcortical regions and attention networks (38). Reduced connectivity between subcortical regions and the DAN in patients with COVID-19, observed in another study, may reflect neuroinflammation (39). At the 3-month follow-up, connectivity had partially recovered, albeit it remained weaker than that in HCs, suggesting a gradual recovery influenced by persistent neuroinflammation (40).

The results of the graph-theoretic analysis showed that *E*_g_ was significantly higher, whereas characteristic path length was significantly lower in patients during the follow-up period relative to in the acute phase; these findings indicate partial restoration of the neural network features. From perceptual and biological perspectives, the overall effectiveness of recovery involves the integration and exchange of information across different brain regions (18). This improvement may enhance the efficiency of tasks, such as attention, decision-making, and cognitive control. Research suggests that cognitive deficits in some patients with COVID-19 may diminish over time, particularly as brain networks realign after the acute phase (2). A decrease in the characteristic path length indicates an increase in the efficiency of communication between different brain regions, suggesting that the brain network connectivity is being restored, and that information dissemination pathways are becoming more concise and efficient. This implies that, after acute inflammation and injury, the brains of patients in the follow-up period began to reconfigure their network structure, which is consistent with the plasticity and healing mechanisms of neural networks (30). Furthermore, the acute phase of COVID-19 may lead to widespread neuroinflammation and blood-brain barrier damage, resulting in decreased global efficiency and increased characteristic path lengths (41). However, as inflammation subsides and the brain’s self-repair mechanisms are activated, neuronal reconnection and network remodeling may lead to an increase in *E*_g_ and a decrease in the characteristic path length during the follow-up period. This recovery process suggests that the functions and structures of areas and connections in the brain damaged during the acute phase can gradually be restored to normal over time.

Nodal analysis revealed that the most prominent differential alterations were localized in the frontal, temporal, and parietal lobes, as well as in the insula, consistent with previously identified morphological alterations. Existing research underscores the role of the frontal lobe in executive functions and working memory, with structural modifications in this region potentially affecting decision-making and problem-solving abilities. The temporal lobe is integral to auditory processing and memory, the parietal lobe to spatial orientation and attentional processes, and the insula to interoceptive awareness and emotion regulation. Alterations in these regions may contribute to cognitive impairment observed in patients with COVID-19 (42–44). Our findings are consistent with those of previous studies. In a 3-month MRI follow-up study, Lu et al. (45) reported that reduced GM volume in the frontal and temporal lobes of recovered patients with COVID-19 correlated with cognitive deficits. Similarly, Qin et al. (46) showed that decreased functional connectivity in the parietal and insular lobes was associated with attention and memory impairments. Previous studies have elucidated the role of the temporal lobe in language comprehension and long-term memory formation. This is consistent with the improvements observed in our VFT results, which indicated recovered language proficiency, executive function, and processing speed (47). These findings suggest that morphological adaptations in specific brain regions may facilitate cognitive recovery, thereby highlighting brain plasticity. Our results highlight the critical need for monitoring cognitive function in patients with COVID-19 and providing targeted rehabilitation strategies to alleviate potential long-term neurological sequelae.

In this study, we conducted five neuropsychological tests, among which DSST and VFT showed significant differences over time, with follow-up scores higher than those recorded during the acute phase. The DSST is a classic neuropsychological test that assesses cognitive abilities, such as attention, processing speed, working memory, and executive functioning (48). Our graph theory analysis results indicated that brain network connectivity and information transmission efficiency improved. These enhancements may have facilitated the recovery of brain regions associated with attention and executive function, thereby increasing the DSST scores. This suggests that the patient’s cognitive function, particularly processing speed and attention, improved during the follow-up period, which is likely related to the recovery of brain structure and function (49). The improvement in the VFT scores may be related to the recovery of LN and language-related networks. Word fluency relies on synergistic interactions between multiple brain regions, especially the limbic system and the frontal regions involved in emotional and language processing (50). The decrease in the characteristic path length during the follow-up period suggests more efficient communication between different language-processing regions of the brain, creating a more direct and simplified path for information transfer (51). Neuropsychological test scores showed a consistent recovery trend aligned with changes in the morphological brain network, demonstrating brain plasticity after the acute phase. As brain networks reorganize and information processing efficiency improves, cognitive function also improves (52). This provides important evidence for understanding the long-term recovery process of patients with COVID-19.

This study had certain limitations. First, the small sample size may limit the generalizability of the findings. Second, the analysis relied solely on morphological networks without incorporating multimodal analyses of functional and structural networks. The inclusion of these additional modalities may provide a more comprehensive understanding of brain network reorganization. Third, this study only explores morphological network changes in patients with COVID-19, which limits its generalizability. Finally, the HC group did not undergo neuropsychological testing, limiting the precision of comparisons with the common group. Implementing neuropsychological testing in the HC group is recommended to enhance the validity of future comparative analyses. This study highlighted the short-term impact of COVID-19 on the brain, marked by significant alterations in morphological subnetworks, particularly those linked to cognitive regulation. Subcortical networks (SCNs) played a compensatory role, with significant recovery of FPN-LN connectivity, whereas other networks showed partial restoration. These findings emphasize the plasticity and repair capacity of the brain post-COVID-19 and suggest that FPN-LN connectivity can serve as a neuroimaging marker for cognitive recovery. The incomplete recovery of certain networks and cognitive functions warrants further longitudinal investigations.

## Data Availability

The original contributions presented in the study are included in the article/Supplementary material, further inquiries can be directed to the corresponding author.
