# Supplementary material for: Longitudinal alterations in morphological brain networks and cognitive function in common-type COVID-19: a 3-month follow-up study
Source: Front Neurol. 2025 Apr 15;16:1549195. doi: 10.3389/fneur.2025.1549195 (PMC12037390; doi:10.3389/fneur.2025.1549195)
Supplement: Supplementary file 1 [file Table_1.DOCX]

Supplementary Material, S1

The matched healthy volunteers in this study were obtained from previous research projects as following:

1. Acupuncture for mild cognitive impairment prevention, corresponding clinical trial registration numbers is ChiCTR-IPR-16009144.
2. Transcutaneous vagus nerve stimulation for functional esophageal disorders, corresponding clinical trial registration numbers is ChiCTR2200064646.
3. Transcutaneous vagus nerve stimulation for modulating premenstrual syndrome brain networks and metabolism, corresponding clinical trial registration numbers is ChiCTR1900020642.

All clinical trial registrations could be found on http://www.chictr.org.cn. The data for the first study were collected before the COVID-19 pandemic, and for the second three study, the healthy participants were recruited during the closed-management period of the COVID-19 pandemic in China.
