# Supplementary material for: Longitudinal alterations in morphological brain networks and cognitive function in common-type COVID-19: a 3-month follow-up study
Source: Front Neurol. 2025 Apr 15;16:1549195. doi: 10.3389/fneur.2025.1549195 (PMC12037390; doi:10.3389/fneur.2025.1549195)
Supplement: Supplementary file 2 [file Table_2.DOCX]

**Supplementary Material**

**Table S2. Changes in intra-network and inter-network morphological connectivity strength at baseline and follow-up**

|  | Acute | | 3 Months | | 3 Months-Acute | |
| --- | --- | --- | --- | --- | --- | --- |
|  | T-value | P-value | T-value | P-value | T-value | P-value |
| DMN vs. DMN | 0.185 | *0.913^a^* | 21.5 | *0.001^a^* | -1.46 | *0.390^c^* |
| DMN vs. DAN | 1.440 | *0.237^a^* | -1.94 | *0.107^a^* | -0.97 | *0.580^c^* |
| DMN vs. FPN | 0.150 | *0.914^a^* | -0.73 | *0.560^a^* | -0.93 | *0.580^c^* |
| DMN vs. LN | -1.760 | *0.138^a^* | 0.88 | *0.490^a^* | 2.86 | *0.041^c^* |
| DMN vs. VAN | -0.387 | *0.845^a^* | 3.01 | *0.640^a^* | 0.29 | *0.820^c^* |
| DMN vs. SCN | 0.531 | *0.812^a^* | -0.6 | *0.630^a^* | -0.15 | *0.890^c^* |
| DAN vs. DAN | -2.010 | *0.118^a^* | 13.5 | *0.001^a^* | -0.25 | *0.850^c^* |
| DAN vs. FPN | -0.110 | *0.920^a^* | -1.51 | *0.220^a^* | -0.78 | *0.610^c^* |
| DAN vs. LN | -4.452 | *0.001^a^* | -2.55 | *0.042^a^* | 1.62 | *0.350^c^* |
| DAN vs. VAN | -3.290 | *0.006^a^* | -1.99 | *0.103^a^* | 0.74 | *0.620^c^* |
| DAN vs. SCN | -4.140 | *0.001^a^* | -2.52 | *0.042^a^* | 1.67 | *0.350^c^* |
| FPN vs. FPN | 2.10 | *0.101^a^* | 24.75 | *0.001^a^* | -0.02 | *0.980^c^* |
| FPN vs. LN | -3.270 | *0.006^a^* | -1.058 | *0.410^a^* | 2.9 | *0.042^c^* |
| FPN vs. VAN | -0.770 | *0.660^a^* | 0.297 | *0.790^a^* | 1.26 | *0.450^c^* |
| FPN vs. SCN | -2.260 | *0.085^a^* | -1.23 | *0.340^a^* | 0.89 | *0.580^c^* |
| LN vs. LN | -0.1579 | *0.913^a^* | 20.45 | *0.001^a^* | 3.4 | *0.053^c^* |
| LN vs.VAN | -0.686 | *0.704^a^* | 0.97 | *0.450^a^* | 2.41 | *0.150^c^* |
| LN vs. SCN | 2.176 | *0.095^a^* | 2.92 | *0.017^a^* | 1.58 | *0.350^c^* |
| VAN vs. VAN | -1.920 | *0.121^a^* | 19.99 | *0.001^a^* | 1.89 | *0.350^c^* |
| VAN vs. SCN | -3.770 | *0.003^a^* | -3.49 | *0.005^a^* | -1.11 | *0.530^c^* |
| SCN vs. SCN | 2.528 | *0.048^a^* | 10.27 | *0.001^a^* | 2.05 | *0.270^c^* |

p^a^ denotes the p-value from the two-sample t-test, and p^c^ denotes the p-value of the paired samples t-test.

DAN, dorsal attention network; DMN, default mode network; FPN, frontoparietal network; LN, limbic network; SCN, subcortical network; VAN, ventral attention network
